# Altered protein conformation and lower stability of the dystrophic transforming growth factor beta-induced protein mutants

**Published:** 2013-03-20

**Authors:** Heather L. Grothe, Morgan R. Little, Phayvanh P. Sjogren, Angela A. Chang, Elizabeth F. Nelson, Ching Yuan

**Affiliations:** Department of Ophthalmology and Visual Neurosciences, University of Minnesota Medical School, Minneapolis, MN

## Abstract

**Purpose:**

Transforming growth factor beta-induced protein (TGFBIp) is a widely expressed extracellular matrix protein that plays roles in cell adhesion and migration, differentiation, apoptosis, bone morphogenesis, and carcinogenesis. Mutations of TGFBIp have been linked to stromal corneal dystrophies, a group of protein conformational diseases characterized by abnormal protein aggregations in the cornea. However, the underlying pathogenic mechanism remains elusive due to a lack of insight into the molecular properties of the disease-causing mutants. In the current study, we applied spectroscopic tools to compare the conformation and protein stability of recombinant wild-type (WT) TGFBIp to two dystrophic mutants, R124C and R555W.

**Methods:**

A serum-free expression system was used to produce the recombinant TGFBIp proteins. Fluorescence and far-ultraviolet circular dichroism spectroscopies were used to compare WT and dystrophic mutants under various conditions.

**Results:**

Our results showed that dystrophic mutants were processed differentially by the expressing cells and produced different proteolytic fragment patterns by proteolysis. Intrinsic tryptophan fluorescence studies revealed moderate shifts in the emission maxima and increased quenching by iodide ion of mutant TGFBIp, suggesting a different conformation than WT protein. Denaturation experiments indicated a difference in protein stability between WT and mutant proteins. Under oxidizing conditions, the mutants produced higher 1-anilinonaphthalene-8-sulfonic acid and thioflavin T fluorescence signals than the WT, indicating increased protein unfolding and fibril formation, respectively. Finally, far-ultraviolet circular dichroism spectroscopy revealed that WT TGFBIp undergoes concentration-dependent conformational changes; similar experiments were not possible on mutant TGFBIp, which remained soluble only at low concentrations.

**Conclusions:**

Our study provides new evidence for the pathogenic mechanism of dystrophic mutants. Although mutant TGFBIp has moderate but consistent structural perturbations, other factors such as oxidation or degradation may be required to cause the phenotypic abnormal aggregations.

## Introduction

The transforming growth factor beta-induced (*TGFBI*) gene, which was first identified from a cDNA library screening effort in TGFβ-1-treated A549 cells, encodes a secretory extracellular matrix protein (TGFBIp) containing 683 amino acids [1]. The primary sequence analysis reveals that TGFBIp contains an N-terminal signal peptide, a cysteine-rich domain (EMI domain in EMILIN), four fasciclin-1 like (FAS-1) domains, and a C-terminal RGD motif. TGFBIp has been shown to be expressed widely by various tissues and cell lines [1-3], and is implicated in cellular functions such as cell attachment, migration, and differentiation [2,3], as well as in pathological events such as tumorigenesis [4-6] or amyloidosis in the cornea [7].

More than 30 missense mutations of TGFBIp in human chromosome 5q31 have been linked to a group of stromal corneal dystrophies characterized by prominent abnormal deposits in the cornea, as either amyloid fibrils and/or amorphous aggregations, and are occasionally associated with chronic epithelial erosion and inflammation [7,8]. The dystrophic mutations primarily cluster around the first and fourth FAS-1 domains. Surgical procedures such as corneal transplant or phototherapeutic keratoplasty have been applied to treat these corneal dystrophies, but are ineffective in preventing the recurrence of the devastating phenotypes. Without proper interventions or treatments, TGFBI-related corneal dystrophies can lead to vision loss.

Although extensive molecular analyses in patient populations have established the genetic etiology of TGFBI-related corneal dystrophies, the molecular basis of the pathogenic mechanism remains unclear. Two amyloidogenic motifs have been previously identified by our group and other researchers and were proposed to be involved in the phenotypic abnormal protein aggregations [9,10]. Interestingly, these two motifs (spanning residues 110 to 131 and residues 515 to 532) are also located within the first and fourth FAS-1 domains, respectively, where most dystrophic mutations reside. However, the roles of these motifs in phenotypic aggregations in vivo await further investigation. Studies on cultured cells or recombinant dystrophic mutants have not demonstrated significant molecular differences between wild-type (WT) and mutant TGFBIp, and thus in vitro experiments have yet to provide conclusive explanations for in vivo phenotypes. For example, WT and dystrophic mutants expressed in cultured human corneal cells all displayed similar patterns of post-translational proteolytic processing, while distinct degradation patterns and abnormal protein turnover were found in the TGFBIp extracted from dystrophic cornea [3,11-13]. Although TGFBIp has been shown to promote cell adhesion, and constant epithelial erosion has been observed in some dystrophic patients, recombinant WT and dystrophic mutants show no difference in cell adhesion or matrix protein binding assays [3,13]. Extensive TGFBIp deposits can be found in the corneas of patients carrying dystrophic mutations, but this phenotype is rarely found in normal patients (only one case has been reported [14]); in contrast to these in vivo findings, recombinant WT and dystrophic mutant TGFBIp all seem to be aggregation-prone and easily form amyloid fibrils in vitro [13]. The lack of transgenic animals displaying dystrophic corneal phenotypes further prevents the development of effective treatments.

Studies on amyloid diseases have strongly suggested that protein conformational changes and unfolding play significant roles in amyloid fibril formation. Factors such as pH, solvation, denaturants, or oxidation, as well as missense mutations, may cause structural perturbations or unfolding, eventually lead to the abnormal aggregations. We previously characterized recombinant WT TGFBIp using various spectroscopic tools and determined its denaturation profile and factors that modulate conformational changes [15]. In the current study, the protein conformation and stability of WT and two dystrophic mutants, R124C and R555W, were compared using established spectroscopic tools to gain insights into the aggregation mechanism of TGFBIp.

## Methods

### Materials

Co^2+^-NTA resin obtained from Pierce Biotechnology (Rockford, IL) was used to purify the expressed TGFBIp protein via the N-terminal hexa-histidine tag (“(His)_6_ tag”), and Strep-Tactin resins (Novagen; Madison, WI) were used for purification via the C-terminal Strep II tag, respectively. Sequencing grade trypsin was purchased from Promega (Madison, WI). Thioflavin T (ThT), 1-anilinonaphthalene-8-sulfonic acid (ANS), and all other chemical reagents not specified were purchased from Sigma-Aldrich (St. Louis, MO).

### Expression and purification of recombinant transforming growth factor beta-induced protein

The production and purification of TGFBIp was performed according to our previous report [15,16]. Briefly, cDNA containing human TGFBI coding sequence was PCR-amplified from an I.M.A.G.E. clone (Clone ID: 2,957,915; GenBank: BE206112) and subcloned into pcDNA3.1 (Invitrogen, Carlsbad, CA) and pIRES.puro2 (“*TGFBIpIRES.puro2*”; Clontech, San Diego, CA) for mutagenesis and expression of recombinant protein. As described in our previous report [15], two constructs encoding the full-length TGFBIp (with signal peptide and RGD motif; 683 amino acids) and a C-terminus truncated TGFBIp (with signal peptide but without the RGD motif; 641 amino acids) were used for the expression experiments. The R124C mutant was generated by inserting a synthetic oligonucleotide cassette (coding strand sequence: 5′-GA TCC ACC ACC ACT CAG CTG TAC ACG GAC *TGC* ACG GAG AAG CTG AGG CCT GAG ATG GAG GGG ml-3′) via the BamH I/Apa I sites into a pCDNA3.1 plasmid containing the KpnI/BstX I fragment of the human TGFBI cDNA. The mutation was confirmed with automated sequencing, and the KpnI/BstX I fragment was used to replace the corresponding region in WT TGFBIpIRES.puro2 to produce the recombinant R124C mutant protein. The R555W mutant was generated using a QuikChange Site-Directed Mutagenesis kit (Stratagene, La Jolla, CA) with a PCR primer (coding strand sequence: 5′-CCA CCA AGA GAA *T*GG AGC AGA CTC-3′), and confirmed with sequencing to ensure the integrity of the coding sequence. The expression plasmids were transfected into 293FT cells followed by puromycin (1 μg/ml) selection to establish individual clones that constitutively express TGFBIp [15]. TGFBIp-expressing 293FT cells were propagated in FreeStyle serum-free medium system (Invitrogen) to produce recombinant proteins, which were then purified with Co^2+^-NTA chromatography. Since TGFBIp has been reported to be aggregation-prone [17], and because we observed this phenomenon in our laboratory, the purified recombinant proteins were used within 1 week for all experiments. To avoid precipitation and concentration-dependent conformational changes (see Results), the concentrations of eluted TGFBIp were immediately measured with bicinchoninic acid protein assay (Pierce Biotechnology), and the proteins were diluted to approximately 0.1 mg/ml. Following dilution, the proteins were dialyzed against Tris-buffered saline (1×TBS, 50 mM Tris-HCl, 150 mM NaCl, pH 7.4) at 4 °C overnight. Dialyzed samples were used for experiments unless otherwise indicated. For concentration-dependent far-ultraviolet circular dichroism (far-UV CD) spectroscopy, WT TGFBIp was further concentrated using a Centriprep YM-30 filter unit (Amicon/Millipore, Billerica, MA) in various concentrations. R124C and R555W precipitated readily during the concentration process and could not be concentrated beyond 0.2 mg/ml.

### Proteolysis of transforming growth factor beta-induced protein with trypsin and chymotrypsin

Purified TGFBIp samples (0.1 mg/ml) in 1×TBS were mixed with trypsin (1:50, enzyme to protein mass ratio) or chymotrypsin (1:12,500) and incubated at room temperature for various durations. A protease inhibitor cocktail was added to terminate the reaction. The digested products were run on sodium dodecyl sulfate–polyacrylamide gel electrophoresis gels and stained with Coomassie Brilliant Blue R-250 for analysis.

### Circular dichroism spectroscopy

The circular dichroism spectra of the TGFBIp samples at various concentrations (WT: 0.1–3.2 mg/ml, R124C and R555W: 0.1 mg/ml) were measured at room temperature using a Jasco J-710 spectropolarimeter (Japan Spectroscopic Co., Tokyo, Japan). Ten traces were averaged for each spectrum, and a binomial smoothing routine provided by the manufacturer was applied.

### Intrinsic tryptophan fluorescence spectroscopy and urea and guanidine hydrochloride denaturation experiments

The intrinsic fluorescence spectra of TGFBIp were measured in a FluoroMax-II spectrofluorometer (Jobin Yvon-SPEX, Edison, NJ) as described in our previous report [15]. The excitation wavelength was 295 nm for tryptophan or 280 nm for aromatic residues, and the emission spectra were scanned from 300 to 500 nm. For each TGFBIp, the emission maxima from three separate batches were measured in the presence of denaturants (urea and guanidine hydrochloride [GndHCl]) at varying concentrations, and the means were plotted against the concentration of denaturant. The half-transition concentration of GndHCl for each protein was determined with curve-fitting using Kaleidagraph software (Synergy Software, Reading, PA). Fluorescence quenching experiments with iodide (I^-^) were also performed as previously described [15]. Aliquots of potassium iodide stock solution (5 M) were sequentially added into a 1 cm quartz cuvette containing TGFBIp protein samples at 25 °C while stirring. To prevent the formation of I_3_- (yellowish color) that interferes with the spectroscopic measurement, sodium thiosulphate was added to the potassium iodide solution at final concentration of 0.1 mM. The emission intensities at 329 nm for WT and 334 nm for R124C and R555W with quencher (F) or without the quencher (F_o_) were determined, and the values were corrected for the dilution factor and the inner filtering effect accordingly [15,18,19]. The ratio of F_o_ to F (F_o_/F) was plotted against the quencher concentration to show the quenching effect of I^-^.

### 1-anilinonaphthalene-8-sulfonic acid and thioflavin T fluorescence spectroscopies

TGFBIp samples were incubated at 37 °C to investigate their stability. For the ANS and ThT assays, 100 μl of samples at 0.1 mg/ml in 1×TBS were mixed with 700 μl of 50 μM ANS (50 mM Tris-HCL, pH 7.4) or 25 μM ThT (50 mM glycine-NaOH, pH 8.5) to scan their fluorescence spectra. The samples were excited at 350 nm and scanned from 400 to 600 nm for the ANS fluorescence, and excited at 450 nm and scanned from 460 to 600 nm for the ThT fluorescence. H_2_O_2_ (0.01%, approximately 3 mM) was also added to the protein samples during incubation to explore the protein stability under oxidizing conditions.

## Results

### Production of recombinant wild-type and mutant transforming growth factor beta-induced protein

**Figure 1:**
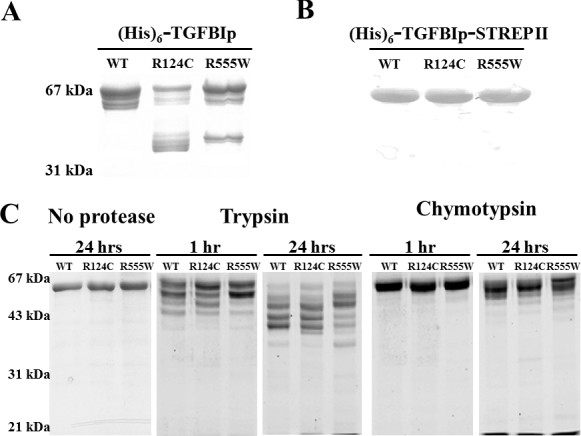
Distinct degradation patterns of recombinant wild-type and mutant transforming growth factor beta-induced protein*.* Recombinant (His)_6_-TGFBIp (**A**) and (His)_6_-TGFBIp-StrepII (**B**) proteins were produced by 293FT cells propagated in FreeStyle serum-free medium system, purified with Co^2+^-NTA chromatography, analyzed on sodium dodecyl sulfate–polyacrylamide gel electrophoresis (SDS–PAGE) gels and stained with Coomassie Brilliant Blue R-250. **C**: Wild-type (WT) and mutant proteins (0.1 mg/ml) from (**B**) were incubated at room temperature either with no protease added as the control, or with trypsin (1:50) or chymotrypsin (1:12,500) for 1 h and 24 h and analyzed with SDS–PAGE gels stained with Coomassie dye. Two μg of samples were loaded for each lane.

We successfully expressed recombinant WT TGFBIp and R124C and R555W dystrophic mutant proteins for our study using a serum-free expression system and two sets of plasmids encoding the full-length protein and the mature, slightly truncated protein, respectively [15]. Although all the recombinant TGFBIp expressed from the full-length constructs displayed multiple protein bands due to extensive post-translational degradation, the R124C and R555W mutants showed distinct degradation patterns compared to those of WT. As shown in Figure 1A, prominent bands around 40 kDa were found in R124C and to a lesser extent in R555W, whereas the majority of protein products in WT were of higher molecular weight (approximately 60 kDa). In contrast, the slightly truncated TGFBIp constructs produced more homogenous protein products that were easily purified to apparent homogeneity with one-step Co^2+^-NTA chromatography (Figure 1B), without the need for multiple rounds of chromatographic separation and purification. Although not necessary for the purification procedure, Strep-Tactin resin was used to confirm the integrity of the C-terminal Strep tag in these recombinant proteins. Consequently, recombinant WT, R124C, and R555W TGFBIp produced from the (His)_6_-TGFBIp-StrepII plasmids were used for the current study. Additionally, all experiments were conducted immediately after the proteins were purified, with concentrations kept at 0.1 mg/ml to prevent aggregation.

### Proteolysis of recombinant wild-type and mutant transforming growth factor beta-induced protein

The results of the proteolysis experiments on the highly purified (His)_6_-TGFBIp-StrepII proteins with trypsin and chymotrypsin, which cleave positively charged residues and aromatic residues, respectively, are shown in Figure 1C. After 1 h of trypsin digestion at 25 °C, R555W displayed a degradation pattern different from that of either WT or R124C TGFBIp. After 24 h of digestion, R124C and R555W produced degradation patterns distinct from WT, with more extensive degradation found in R555W. The differences produced by chymotrypsin digestion were less evident, but R555W again generated more fragments after 24 h of digestion.

### Far-ultraviolet circular dichroism spectra of recombinant wild-type and mutant transforming growth factor beta-induced protein

**Figure 2:**
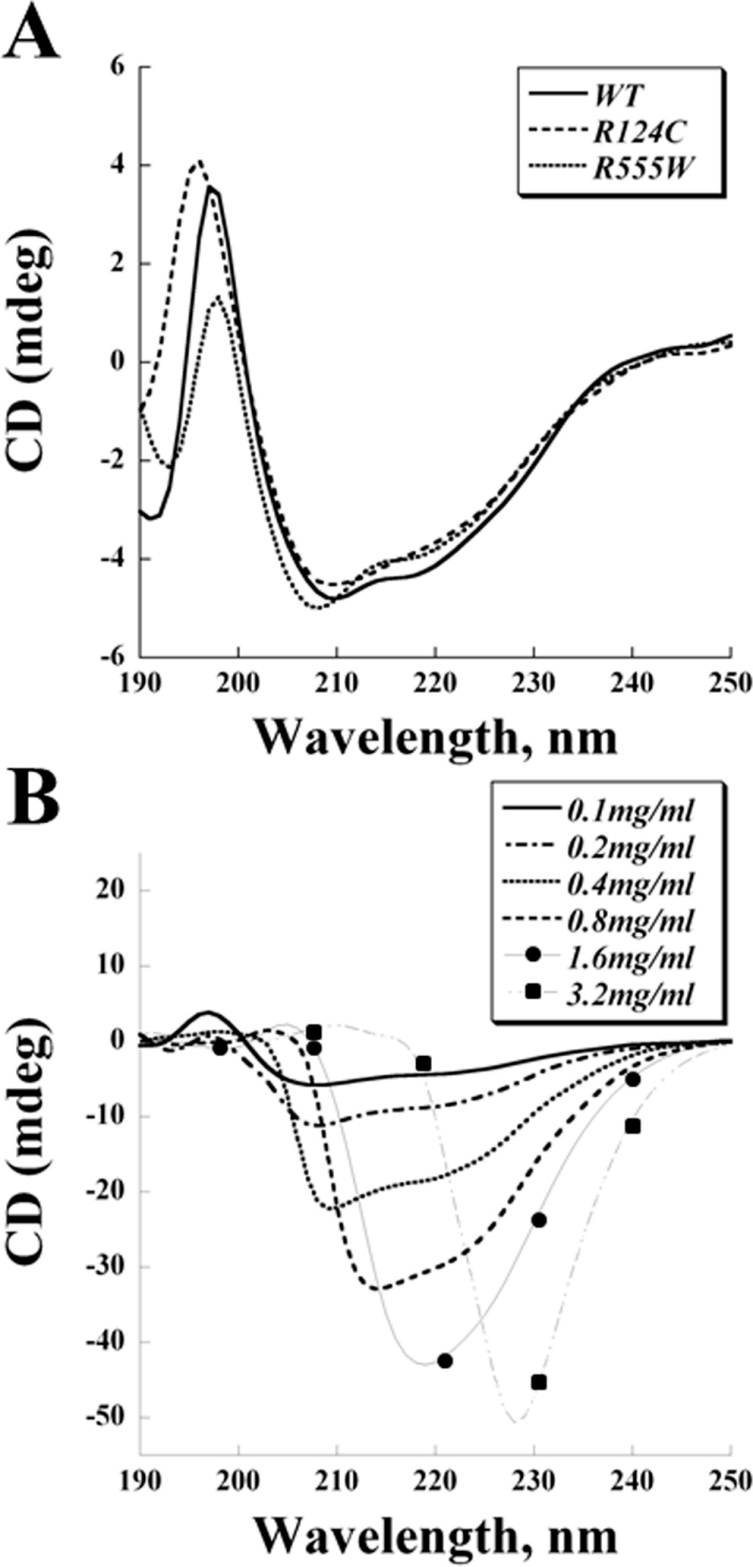
Far-ultraviolet circular dichroism spectra of recombinant wild-type and mutant transforming growth factor beta-induced protein*.* **A**: Far-ultraviolet circular dichroism (far-UV CD) spectra of recombinant transforming growth factor beta-induced protein (TGFBIp). The protein concentrations were 0.1 mg/ml. **B**: The concentration-dependent conformational changes of wild-type (WT) TGFBIp. The far-UV CD spectra of WT TGFBIp were measured at 0.1, 0.2, 0.4, 0.8, 1.6, and 3.2 mg/ml.

Far-UV CD spectroscopy was used to investigate the secondary structure of the recombinant TGFBIp. Deconvolution of the 200–240 nm regions of the spectra with an online tool, k2d, revealed that the secondary structure of each TGFBIp was composed of approximately 32%–34% of α-helices and 10%–12% β-sheets (Figure 2A).

At the protein concentration used for far-UV CD (0.1 mg/ml), we did not observe any signals in the near-UV region (300–325 nm), which detects quaternary structure. To increase the signals in this region, we concentrated the purified recombinant TGFBIp. Attempts to concentrate R124C and R555W beyond 0.2 mg/ml led to protein precipitation and therefore significant sample loss. WT TGFBIp, in contrast, was successfully concentrated to 4 mg/ml. However, a concentration-dependent effect on the far-UV CD spectra was observed, indicating secondary structural changes (Figure 2B). At lower concentrations, WT TGFBIp appeared to be rich in α-helices and β-sheets. Concentrating the protein to 0.8 mg/ml or greater resulted in a global conformational change: The troughs at 208 nm and 222 nm disappeared, and the 218 nm dip became more prominent, signifying the loss of α-helices and an increase in formation of β-sheets, respectively. At 3.2 mg/ml, the WT TGFBIp displayed a prominent 228 nm dip, suggesting β-turns. Diluting the concentrated protein to lower concentrations reversed these results, and the α-helix/β-sheet-rich conformers were again observed.

### Intrinsic tryptophan fluorescence spectra of recombinant wild-type and mutant transforming growth factor beta-induced protein and denaturation profiles

**Figure 3:**
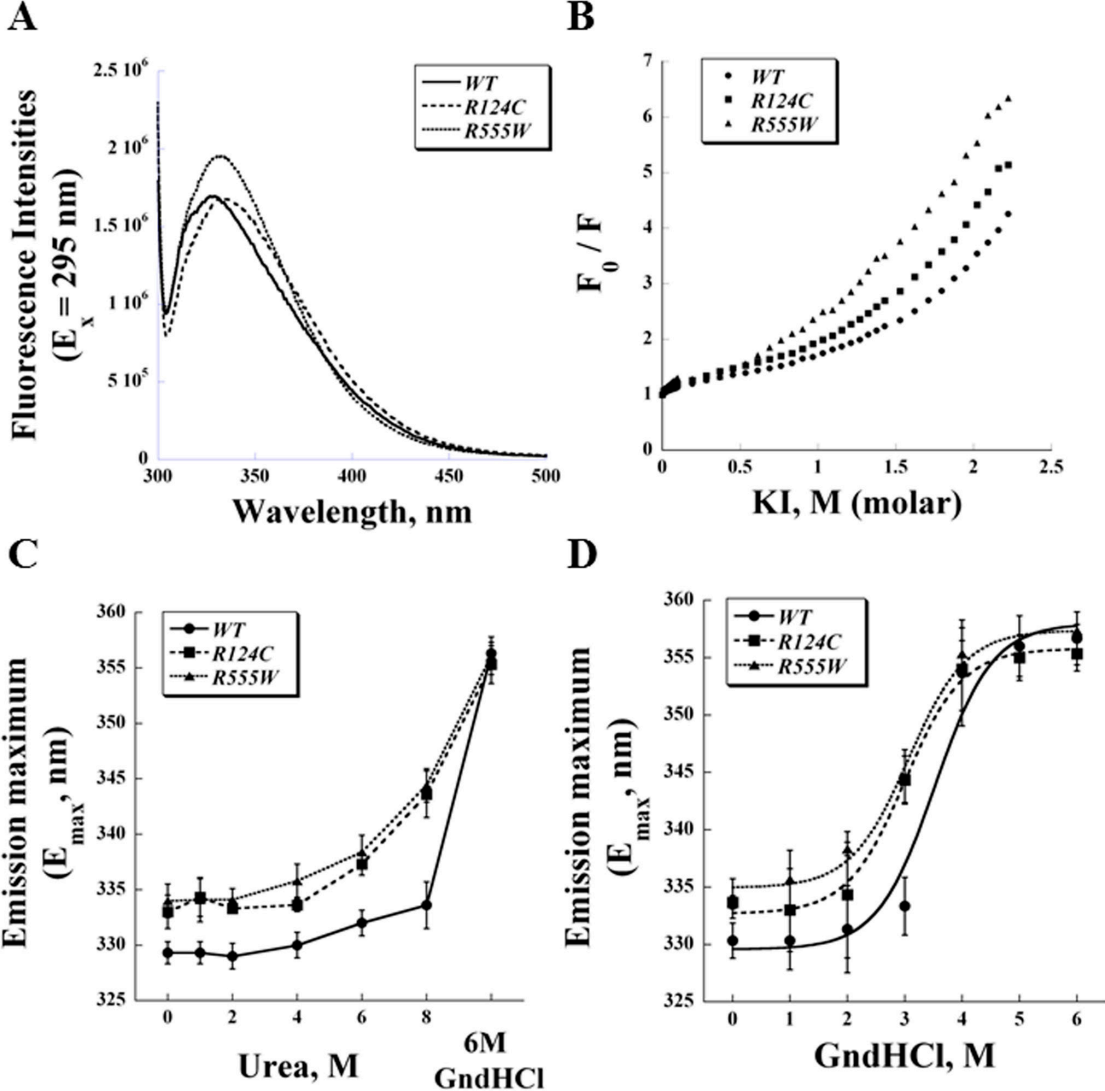
Intrinsic tryptophan fluorescence of recombinant wild-type and mutant transforming growth factor beta-induced protein. **A**: The fluorescence spectra of recombinant transforming growth factor beta-induced protein (TGFBIp; 0.1 mg/ml) excited at 295 nm. The emission maxima are 329 nm for wild-type (WT) and 334 nm for R124C and R555W. **B**: The intrinsic fluorescence of recombinant TGFBIp was quenched using increasing concentrations of iodide (I^-^). The denaturation profiles of recombinant TGFBIp were investigated by (**C**) urea and (**D**) guanidine hydrochloride (GndHCl). The half-transition concentrations of GndHCl were determined to be 3.6±0.1 M for WT, 2.8±0.2 M for R124C, and 3.0±0.1 M R555W. (n=3, bar=standard deviation [SD]).

WT and R124C mutant TGFBIp contain two tryptophan residues, W68 and W148, whereas the R555W mutant protein has an additional tryptophan residue due to the mutation. R555W TGFBIp displayed higher intrinsic tryptophan fluorescence intensity than either WT or R124C TGFBIp (Figure 3A), likely due to its additional tryptophan residue (W555) and/or altered quantum efficiency. The emission maximum of each mutant protein was shifted approximately 5 nm to the red (333.6±0.9 nm for R124C and 333.8±1.9 nm for R555W) compared to that of WT (329.3±0.7 nm; Figure 3A and Table 1).

**Table 1:** Comparison of the molecular properties of WT and mutant TGFBIp.

**Molecular properties**	**WT**	**R124C**	**R555W**
**E_max_**	**329.3±0.7 nm**	**333.6±0.9 nm**	**333.8±1.9 nm**
**Urea resistance** **(E_max_ shift)**	**Yes** **(~4 nm)**	**Partial** **(~10 nm)**	**Partial** **(~10 nm)**
**[GndHCl]_1/2_**	**3.6±0.1 M**	**2.8±0.2 M**	**3.0±0.1 M**
**I^-^ quenching**	**+**	**+ +**	**+ + +**
**Highest concentration**	**4 mg/ml**	**0.2 mg/ml**	**0.2 mg/ml**
**H_2_O_2_-induced increase** **of ANS fluorescence**	**-**	**+**	**+**
**H_2_O_2_-induced increase** **of ThT fluorescence**	**-**	**+**	**+**

E_max_: the emission maximum of the intrinsic tryptophan fluorescence. [GndHCl]_1/2_: the half-transition concentration of GndHCl. Highest concentration: maximum concentration before protein precipitates from solution.

Because a red shift in tryptophan fluorescence may indicate exposure of the residue to solvent, we performed quenching experiments to compare this aspect of conformation between WT TGFBIp and the dystrophic mutant proteins. Aqueous I^-^ was added in increasing concentrations from 0 to 2.2 M, and the ratio of fluorescence intensity in the absence of quencher to the intensity in the presence of quencher was determined (Figure 3B, “F_0_/F”). At concentrations of 1 M or less, I^-^ did not significantly quench the tryptophan fluorescence of WT; in contrast, these low concentrations had a slightly greater quenching effect on R124 protein and notably decreased the tryptophan fluorescence of R555W protein. At higher I^-^ concentrations, the tryptophan fluorescence of both mutant proteins was more prominently reduced than that of the WT protein (Figure 3B).

In our previous report, we established the denaturation profile of WT TGFBIp according to intrinsic fluorescence; we used the same method here to investigate and compare potential perturbations in the conformations of the dystrophic mutants [15]. Tryptophan fluorescence spectra confirmed that WT TGFBIp was relatively resistant to denaturation by urea at concentrations up to 8 M (Figure 3C; see also reference [15]); whereas R124C and R555W TGFBIp were partially unfolded by urea, as indicated by the shift in their tryptophan fluorescence approximately 10 nm toward the red. Six molar guanidine hydrochloride (GndHCl) denatured all three TGFBIp proteins (Figure 3C, “6M GndHCl”) and caused a red shift in their emission maxima to approximately 355 nm. We also performed intrinsic fluorescence spectroscopy of WT and mutants TGFBIp using 280 nm to excite all aromatic residues instead of just the tryptophan residues, and obtained similar urea resistance results (data not shown). Further investigation of GndHCl denaturation demonstrated that the half-transition occurred at a higher concentration of denaturant for WT TGFBIp (3.6±0.1 M) than for either R124C (2.8±0.2 M) or R555W (3.0±0.1 M) mutant TGFBIp (Figure 3D and Table 1).

### 1-anilinonaphthalene-8-sulfonic acid and thioflavin T fluorescence spectra of recombinant wild-type and mutant transforming growth factor beta-induced protein

To compare the protein stability of the WT and mutant TGFBIp, we used ANS as an indicator for protein unfolding and ThT as an indicator of fibril formation. ANS binds to exposed hydrophobic regions of proteins and as a result fluoresces to a much higher degree. Similarly, ThT fluorescence intensity increases as the dye binds to amyloid fibrils.

**Figure 4:**
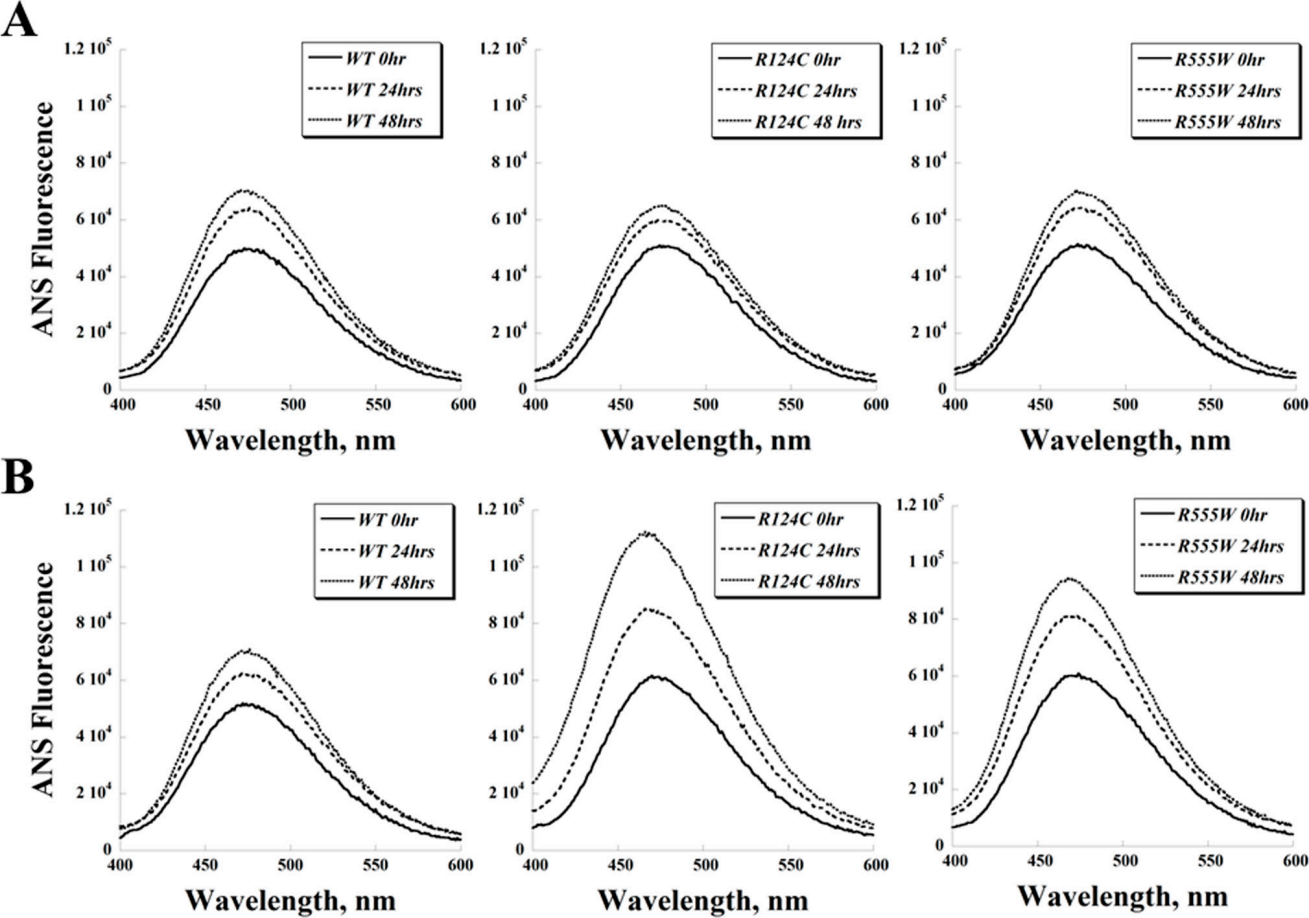
1-anilinonaphthalene-8-sulfonic acid fluorescence spectra of recombinant wild-type and mutant transforming growth factor beta-induced protein. **A**: The 1-anilinonaphthalene-8-sulfonic acid (ANS) fluorescence spectra of wild-type (WT), R124C, and R555W proteins incubated at 37 °C for various lengths of time. **B**: H_2_O_2_ (0.01%) was also added to the samples at the beginning of the incubation to investigate the effect of oxidation on the conformation of transforming growth factor beta-induced protein (TGFBIp).

The baseline ANS fluorescence intensities of R124C and R555W TGFBIp were comparable to that of WT TGFBIp (Figure 4A, “0hr”), and incubation at 37 °C for 24 or 48 h resulted in only limited increases of 25%–40% (Figure 4A). To investigate the protein stability under oxidative conditions, H_2_O_2_ was added at the beginning of incubation. At 24 and 48 h, the ANS fluorescence intensities of WT TGFBIp in the presence of H_2_O_2_ were similar to the intensities of the protein at those time points without the oxidizing agent (Figure 4A,B). In contrast, R124C and R555W TGFBIp produced higher ANS fluorescence signals when H_2_O_2_ was added during incubation (Figure 4A,B).

**Figure 5:**
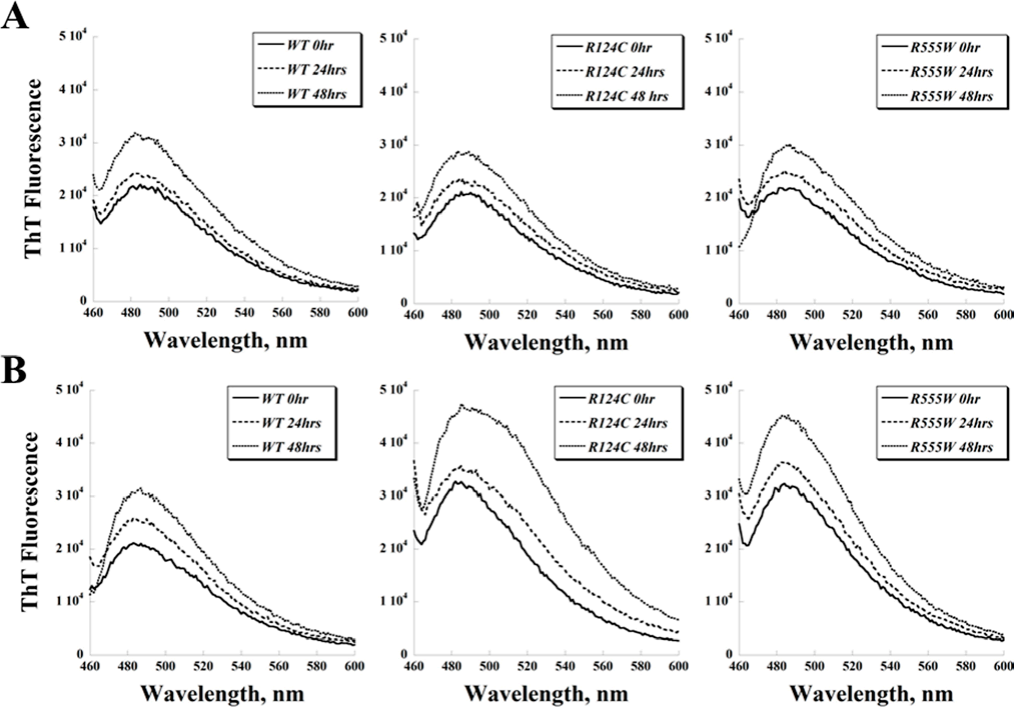
Thioflavin T fluorescence spectra of recombinant wild-type and mutant transforming growth factor beta-induced protein*.* **A**: The thioflavin T (ThT) fluorescence spectra of wild-type (WT), R124C, and R555W proteins incubated at 37 °C for various lengths of time. **B**: H_2_O_2_ (0.01%) was also added to the samples at the beginning of the incubation to investigate the effect of oxidation on the fibril formation of transforming growth factor beta-induced protein (TGFBIp).

The ThT fluorescence spectra paralleled the ANS results: WT, R124C, and R555W TGFBIp showed similar trends of moderately increased ThT fluorescence after 24 and 48 h of incubation at 37 °C (Figure 5A). The ThT fluorescence intensities of the WT protein in the presence of H_2_O_2_ (Figure 5B) were similar to those in the absence of the oxidizing agent (Figure 5A). The addition of H_2_O_2_ led to higher ThT signals for the R124C and R555W proteins at all three time points measured (Figure 5B).

## Discussion

In the current study, we investigated recombinant WT TGFBIp and two dystrophic mutants, R124C and R555W, with spectroscopic methods. R124C and R555W were selected because these two mutations represent distinct phenotypes of corneal dystrophies: The R124C mutation is associated with type I lattice dystrophies, and R555W, with type I granular dystrophies. Although previous studies have established the genetic etiologies of TGFBIp-related corneal dystrophies, how these genetic mutations alter the molecular properties of TGFBIp and result in extensive protein aggregation or amyloid fibril formation remains unclear [7,8]. Various researchers have performed comparison studies of recombinant WT and mutant TGFBIp in hopes of identifying molecular differences that will correlate with the observed dystrophic phenotypes; unfortunately, these investigations have yet to yield conclusive evidence and often have reported no differences between the WT and mutant recombinant proteins. For example, WT and dystrophic mutants expressed by Chinese hamster ovary cells and corneal epithelial cells display identical degradation patterns regardless of mutations, readily form amyloid fibrils in vitro, and bind the extracellular matrix and facilitate cell attachment equally [3,12,13]. More recently, Runager et al. were the first to demonstrate that a recombinant mutant TGFBIp, A546T, is less stable than recombinant WT protein by using transverse urea gradient gel electrophoresis [20]. In the same report, however, the denaturation profiles of other mutant proteins, for example, R124C, indicated similar stability to the WT protein, and the profiles of some mutants, including R555W, indicated greater stability than the WT protein. The authors proposed the dystrophic phenotypes may be attributed to the lower stability of the fourth FAS-1 domain for mutants with mutations within this domain; however, for mutants with mutations outside the fourth FAS-1 domain (such as R124C), the pathogenic mechanism remains unclear.

Similar to other researchers, we examined the post-translational proteolytic processing of recombinant WT and mutant TGFBIp. TGFBIp from tissues and cultured cells is typically characterized by an extensive post-translational degradation pattern when analyzed with sodium dodecyl sulfate–polyacrylamide gel electrophoresis gels [3,11-13,15,20,21]. In this study, we showed distinct post-translational degradation patterns between the WT and dystrophic mutant proteins, when full-length WT and mutant constructs were used (Figure 1A), using a serum-free culture system. Although mature full-length TGFBIp is found in corneal tissues, the predominant isoform in the cornea was truncated at the C-terminus [22]. Additionally, researchers have reported that the RGD motif is cleaved or absent completely from TGFBIp produced by cultured Chinese hamster ovary cells [20,23]. We discovered that removing the RGD motif significantly reduced proteolytic degradation and facilitated the production of a predominant protein product. This slightly truncated form of TGFBIp was simpler to purify to homogeneity (Figure 1B), and was used in our previous study of WT TGFBIp [15], as well as in the current comparison study of WT and mutant proteins.

A recent study [11] found differential degradation and accumulation of distinct peptide fragments of TGFBIp extracted from the dystrophic corneas, and proposed that the abnormal turnover of mutant TGFBIp in the cornea may contribute to the dystrophic phenotypes. In our current study, the differential degradation pattern between the WT and dystrophic mutants was also generated by proteolysis of purified TGFBIp (Figure 1C), indicating that the mutants may assume different conformations with altered accessibility of potential protease cleavage sites. However, because arginine is one of the substrates for trypsin, a mutation at an arginine residue (specifically residue 124 and residue 555 for the mutants in our study) may simply eliminate one of the potential trypsin digestion sites, resulting in the observed proteolytic products. Similarly, chymotrypsin cleaves peptides at aromatic residues, and thus, the additional tryptophan residue in the R555W mutant may provide another digestion site for chymotrypsin. Nevertheless, due to either altered conformation or altered protease digestion sites, our in vitro degradation results corroborate with the in vivo findings [11] and suggest a potential pathological link between mutant protein fragmentation and dystrophic phenotypes. In addition, as putative amyloidogenic motifs have been found on TGFBIp [9,10], accumulation of fragments containing these amyloidogenic motifs may lead to abnormal amyloid fibril deposits in the dystrophic corneas. TGFBIp in the cornea may be degraded by a different protease(s) than those used in our current experiments. A recent study identified Htr-A1 protease in the amyloid deposits of dystrophic corneas and suggested it may be involved in the turnover of TGFBIp [24].

Intrinsic tryptophan fluorescence spectroscopy can be sensitive to localized perturbations of the microenvironment and thus serves as an indicator of either local or global protein unfolding. The intrinsic tryptophan fluorescence profiles of the three recombinant TGFBIp provide further evidence that the two mutant proteins have different conformations than the WT protein, as indicated by the moderate red shift in the emission maxima of R124C and R555W proteins (Figure 3A). Because this shift suggests partial exposure of tryptophan to the aqueous solvent, we performed quenching experiments using I^-^ as an ionic quencher and showed that the tryptophan fluorescence of both mutant TGFBIp can indeed be quenched to a greater extent than that of WT TGFBIp (Figure 3B).

Although results from the intrinsic fluorescence spectroscopy indicate structural perturbations caused by these dystrophic mutations, these conformational aberrations seemingly do not induce significant global secondary structural changes reflected by far-UV CD spectroscopy. Previous CD spectroscopy studies on the fourth FAS-1 domains of TGFBIp revealed no distinct features between WT and dystrophic mutants despite their difference in protein stability [19]. Our current far-UV CD spectra also showed that WT, R124C, and R555W TGFBIp contain similar secondary structure compositions (Figure 2A). However, we observed concentration-dependent conformational changes in the secondary structure of the WT protein (Figure 2B). WT TGFBIp transitioned from a conformer containing α-helices and β-sheets at lower protein concentrations to a conformer composed primarily of β-sheets at 0.8–1.6 mg/ml. At the highest concentration examined (3.2 mg/ml), the WT protein transformed into a conformer with a dominant trough near 230 nm (specifically, at 228 nm). Concentration-dependent conformational changes have been observed for various proteins. For example, a molten globular state of histones can be induced either by increasing protein concentrations or by adding macromolecular crowding agents, such as Ficoll or polyethylene glycol [25,26]. Although the identity of the 230 nm species awaits further investigation, this negative peak was also reported in a spectroscopic study on denatured concanavalin A and pea lectin I, and has been proposed to be the defining characteristic of β-turns [27-29]. Alternatively, this intriguing 230 nm species may be partially attributed to tryptophan residues, as in the case of α-chymotrypsinogen activation, or may be caused by altered hydrophobic interactions, as observed in certain dihydrofolate reductase mutants [30,31]. Since the conformation of WT TGFBIp appears to be concentration-dependent, biophysical approaches that require high protein concentrations, including Fourier transformed infrared spectroscopy, near-UV CD spectroscopy, nuclear magnetic resonance spectroscopy, or X-ray crystallography, may not be suitable for structure and conformation studies. Additionally, although a recent study [20] of TGFBIp produced denaturation profiles of WT and mutant TGFBIp using transverse urea gradient gel electrophoresis, because the compression of protein bands during electrophoresis increases the local protein concentration, this approach may also cause deviation from the native folding of TGFBIp if concentration-dependent conformational changes occur.

We did not obtain far-UV CD spectra of concentrated R124C and R555W proteins because neither could be concentrated beyond 0.2 mg/ml without precipitating extensively; others have reported the instability of mutant TGFBIp [17]. Therefore, whether R124C and R555W TGFBIp experience conformational changes at higher concentrations as does the WT protein remains unclear. However, the fact that both mutant proteins so readily precipitated supports the hypotheses that the proteins’ conformations differ, albeit subtly, from that of the WT protein and their altered structures may render them more susceptible to concentration-dependent conformational changes. Although lower stability of TGFBIp mutants at higher protein concentrations may also contribute to the phenotypic aggregations in dystrophic corneas, the in situ concentration of TGFBIp has yet to be determined and may depend on the turnover mechanism in the cornea and whether the protein is sequestered by binding covalently or noncovalently to collagen fibrils [22].

Structural perturbations may destabilize a protein, potentially leading to protein unfolding and the pathogenic aggregations characteristic of various amyloid diseases [32]. Protein unfolding and conformational changes can result in the exposure of intrinsic amyloidogenic regions, which are essential for the formation of abnormal aggregations or of amyloidogenic intermediates [32]. For example, in the amyloidosis of human transthyretin, the sequence-dependent unfolding pathway in these mutants may lead to the production of amyloidogenic intermediates, even though only minor structural differences between WT and disease-associated mutant proteins were found [33]. Our current study has demonstrated the lower stability of R124C and R555W mutants compared to the WT. The dystrophic mutants were more susceptible to urea denaturation, as indicated by greater red shifts in the emission maxima of their intrinsic tryptophan fluorescence (Figure 3C), and both mutant proteins were unfolded by lower GndHCl concentrations than was the WT protein (Figure 3D). The distinct denaturation profiles in mutant TGFBIp suggest unique unfolding processes may potentially be the key for understanding the pathogenic mechanism of TGFBIp-related corneal dystrophies.

We used ANS fluorescence spectroscopy and ThT fluorescence spectroscopy to investigate the conformational changes of WT and mutant TGFBIp further, specifically to examine protein unfolding and the formation of amyloid fibrils, respectively. Because protein unfolding and amyloid fibril formation can be modulated by various factors, we tested the effect of oxidation on the formation of amyloid fibrils by TGFBIp. The increase in ANS signal intensity from 0 h to 24 and 48 h was similar for each TGFBIp when incubated at 37 °C (Figure 4A). Next, we examined the effect of an oxidizing agent, H_2_O_2_, on the three proteins. At the H_2_O_2_ concentration used in our study (0.01%, approximately 3 mM), methionine and tryptophan residues are oxidized [34]; other residues such as cysteine can be as well. Since TGFBIp contains a cysteine-rich domain (EMI domain in EMILIN), oxidants can cause the formation of non-native disulfide bonds and consequently alter the conformation and stability of the protein. When H_2_O_2_ was added to the incubations, both dystrophic mutant proteins produced significantly higher ANS fluorescence signals, indicating protein unfolding, whereas the WT protein appeared to be relatively resistant to oxidizing conditions for up to 48 h (Figure 4B). The ThT fluorescence experiments showed results similar to the ANS fluorescence studies (Figure 5), which indicate a correlation between protein unfolding and fibril formation. The results of our ANS and ThT experiments also suggest that factors, such as oxidation, may be essential to the mechanism causing pathogenic aggregations in the cornea.

In summary, our current study demonstrates moderate yet consistent structural differences between the WT TGFBIp and mutant proteins; and we propose that these differences may be amplified by extrinsic conditions to promote protein instability, unfolding, and ultimately dystrophic aggregations of mutant TGFBIp. Since numerous degradation products are present in the cornea and because the majority of the literature reveals few molecular differences between full-length WT and mutant TGFBIp, comprehensive studies of truncated fragments are warranted. Further investigation of the slightly truncated recombinant TGFBIp we generated and other fragments may help determine the role of individual degraded fragments in the development of phenotypic aggregations. Finally, we demonstrated that R124C and R555W mutant TGFBIp are less stable than the WT protein in the presence of denaturants and an oxidant. Since fragmentation and various factors, such as oxidation, pH, and solvation, have been proposed to cause amyloid fibrils and amorphous protein aggregations, characterization of these recombinant TGFBIp proteins under conditions relevant to the unique physiology of cornea may help clarify the process by which mutant TGFBIp causes these dystrophic phenotypes.
